# Prevalence and Associated Factors of Latent Tuberculosis Infection Among Healthcare Workers in a Mexican Tertiary Care Hospital

**DOI:** 10.3390/diseases13060173

**Published:** 2025-05-30

**Authors:** José Ángel Hernández-Mariano, Mónica Alethia Cureño-Díaz, Verónica Fernández-Sánchez, Estibeyesbo Said Plascencia-Nieto, Dulce Milagros Razo-Blanco-Hernández, Claudia Vázquez-Zamora, Víctor Hugo Gutiérrez-Muñoz, Beatriz Leal-Escobar, Erika Gómez-Zamora, Yanelly Estrella Morales-Vargas

**Affiliations:** 1 Department of Research, Hospital Juarez of Mexico, Mexico City 07760, Mexico; 2 Direction of Medical Teaching and Research, Hospital Juarez of Mexico, Mexico City 07760, Mexico; 3 Postgraduate Studies and Research Section, School of Medicine, National Polytechnic Institute, Mexico City 07738, Mexico; 4 Department of Infectious Disease, Hospital Juarez of Mexico, Mexico City 07760, Mexico; 5 Epidemiological Surveillance Unit, Hospital Juarez of Mexico, Mexico City 07760, Mexico; 6 Department of Medical Management, Hospital Juarez of Mexico, Mexico City 07760, Mexico; 7 School of Medicine, National Autonomous University of Mexico, Mexico City 04510, Mexico

**Keywords:** health personnel, healthcare workers, latent tuberculosis, mycobacterium tuberculosis infection

## Abstract

Background/Objectives: Healthcare workers (HCWs) are globally recognized as a high-risk group for tuberculosis (TB) infection. However, limited data exist on the prevalence of latent TB infection (LTBI) and associated occupational risk factors in the Mexican context. Identifying the burden of LTBI is essential for effective prevention. This study aimed to estimate the prevalence of LTBI among HCWs in a tertiary care hospital in Mexico and to explore associated risk factors. Methods: An analytical cross-sectional study was conducted among 300 HCWs (including physicians, nurses, and stretcher-bearers) at a tertiary-level hospital in Mexico. Sociodemographic and occupational data were collected through a structured questionnaire. LTBI screening was performed using the tuberculin skin test (TST), with positive results confirmed via the QuantiFERON-TB Gold assay. Associations between relevant variables and LTBI were assessed using logistic regression models, adjusted for potential confounders. Results: The prevalence of LTBI was 16.7%. After adjusting for confounders, male HCWs had significantly higher odds of LTBI compared to females (adjusted odds ratio [aOR] = 2.02; 95% confidence interval [CI]: 1.06–3.80). Although elevated odds of LTBI were also observed among physicians, stretcher-bearers, and those with direct contact with TB patients, these associations were not statistically significant. Conclusion: LTBI represents a relevant occupational health issue among HCWs, with nearly one in six workers affected. Early detection and prevention of TB in healthcare settings are critical to protecting individual workers and public health. These findings highlight the need to strengthen occupational TB surveillance and prevention strategies in similar healthcare environments.

## 1. Introduction

In 2023, an estimated 11 million individuals worldwide developed tuberculosis (TB), and approximately 1.25 million died from the disease. These figures reestablished TB as the leading cause of death from a single infectious agent, following a three-year period during which COVID-19 surpassed it. In the Region of the Americas alone, 325,000 new TB cases and 35,000 TB-related deaths were reported in the same year, representing increases of 14% and 41%, respectively, compared to 2015 data [1]. Mexico is classified as a country with a moderate incidence of TB within the Region of the Americas, reporting approximately 19,000 new cases and over 2000 TB-related deaths annually [2].

TB cases are classified as either latent TB infection (LTBI) or active TB disease. LTBI is a persistent immune response to *Mycobacterium tuberculosis (MTB*) antigen stimulation, without clinical or radiological evidence of active disease. In contrast, active TB is characterized by clinical symptoms resulting from an ongoing *MTB* infection, which can affect various organs, although pulmonary TB is the most common and infectious form. It is estimated that approximately 5% to 15% of individuals with LTBI will progress to active TB during their lifetime, with the highest risk occurring within the first two years following initial infection [3].

The BCG (Bacille Calmette–Guérin) vaccine is administered in Mexico as part of the National Immunization Program. It is free of charge and primarily targeted at newborns to prevent severe forms of TB, such as tuberculous meningitis and miliary TB [4,5]. The vaccine is not routinely administered to adults for three main reasons. First, its effectiveness in preventing pulmonary tuberculosis (the most common and contagious form in adults) is limited and variable. Second, vaccinating an adult already immunized in childhood does not offer significant additional protection, as the vaccine’s protective effect decreases over time. Third, the BCG vaccine interferes with interpreting the tuberculin skin test (TST) by inducing false positives due to cross-reactivity with vaccine strain antigens.

Due to their continuous occupational exposure to *MTB*, healthcare workers (HCWs) are at increased risk of both LTBI and progression to active TB disease, particularly in regions with high TB prevalence. This endangers their health and poses a risk of transmission to patients and coworkers [6,7]. The risk of LTBI among HCWs is influenced by multiple factors, including the local incidence of TB, structural and operational characteristics of healthcare facilities, the nature and frequency of clinical activities performed, and the implementation of disease prevention and control measures [8,9,10].

Although several studies have investigated TB infection among HCWs [8,9], there remains a lack of clear information on its frequency in the Mexican context and the associated risk factors in workplace settings. Understanding the burden of LTBI among HCWs is essential for developing and implementing effective TB control strategies within healthcare facilities. Hence, this study aimed to determine the prevalence of LTBI among HCWs in a tertiary care hospital in Mexico and identify associated risk factors.

## 2. Materials and Methods

### 2.1. Design and Study Population

An analytical cross-sectional study was conducted between June and August 2023 in a tertiary hospital in Mexico City, Mexico. Study participants were physicians, nurses, and stretcher-bearers aged 18 years or older who had worked for at least two years at the hospital where this study was conducted. Immunosuppressed individuals with a history of allergic reactions to tuberculin tests and pregnant women were excluded. Of the 534 HCWs who met the eligibility criteria, 300 agreed to participate in this study (response rate = 56.2%). Based on the sample size, this study’s statistical power was estimated to assess the associations of interest. For this purpose, one of the independent variables (i.e., sex) was selected as the reference. According to previous literature, 54% of female healthcare workers tested negative for LTBI [9]. Under this assumption, this study was determined to have 80% power to detect an odds ratio of at least 1.6, with a significance level of 95%.

### 2.2. Data Collection

Sociodemographic and employment information were collected using a structured questionnaire. LTBI was initially assessed using the TST. Participants received an intradermal injection of 0.1 mL of purified protein derivative (PPD) tuberculin on the volar surface of the forearm, following the Mantoux technique. A TST result was considered positive if the induration measured more than 10 mm in diameter. The QuantiFERON-TB Gold (QFT) assay was subsequently performed to confirm TB infection. Participants with an interferon-gamma (IFN-γ) response of ≥0.35 IU/mL following antigen stimulation were considered positive for latent TB.

### 2.3. Statistical Analysis

The study variables were described using frequencies and percentages. Sociodemographic and employment characteristics were compared according to the presence of latent TB using the two-tailed Pearson chi-squared test. Logistic regression models were constructed to identify factors associated with latent TB. All models were adjusted for potential confounding variables. Based on biological plausibility, sex and age were included in all final models as a priori confounders. Additional variables were evaluated for confounding using the change-in-estimate criterion; variables were retained if their removal resulted in a change of ≥10% in the OR for the primary association. This was conducted in a stepwise backward elimination process, beginning with all candidate variables in the model and removing them one at a time.

Statistical significance for all models was based on a *p*-value < 0.05. All analyses were performed using the STATA statistical package, version 15.1 (Stata Corporation, College Station, TX, USA).

## 3. Results

The prevalence of LTBI among participants was 16.7% (95% confidence interval [CI]: 13–21%). The study population was predominantly female (55.6%), aged 30 years or older (69.3%), and composed mainly of nurses (77.7%). Additionally, 67.3% of participants had five years or less of professional experience at the hospital. Most participants were working in inpatient care units at the time of this study (87.3%), and 24.0% reported previous contact with patients diagnosed with active tuberculosis. When compared to HCWs without LTBI, those with the infection were more likely to be male. No statistically significant differences were observed in the other sociodemographic or occupational variables (Table 1).

**Figure 1:**
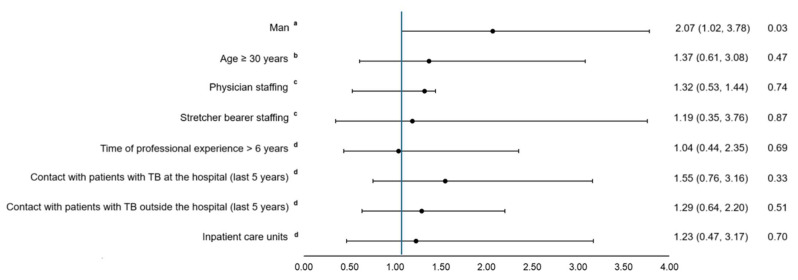
Logistic regression analysis of factors associated with LTBI. ^a^ Adjusted by age, time of professional experience, and hospital unit. ^b^ Adjusted by sex, time of professional experience, and hospital unit. ^c^ Adjusted by sex, age, time of professional experience, and hospital unit. ^d^ Adjusted by sex and age.

After adjusting for potential confounders, men compared with women had higher odds of having LTBI (adjusted OR [aOR] = 2.02; confidence interval [95% CI] = 1.06, 3.80). Physicians (aOR = 1.32; 95% CI: 0.53–4.44) and stretcher-bearers (aOR = 1.19; 95% CI: 0.35–3.76) also showed higher odds of LTBI than nurses, although these associations were not statistically significant. Similarly, being older than 30 years (aOR = 1.37; 95% CI: 0.61–3.08), having had contact with TB patients (aOR = 1.55; 95% CI: 0.76–3.16), and working in inpatient care units (aOR = 1.23; 95% CI: 0.47–3.17) were associated with increased odds of LTBI, but these associations did not reach statistical significance (Figure 1). Similar associations were observed in crude models (Supplementary Material S1).

## 4. Discussion

In our study, 16.7% of the 300 HCWs tested positive for LTBI. These findings were consistent with the rate reported by a meta-analysis integrating data from 38 studies conducted among HCWs in Latin America and the Caribbean (17%; IC 95% = 9.0, 25%) [10]. Although Mexico is considered a country with a moderate incidence of TB, the distribution of the disease is not uniform across the national territory. Mexican states bordering the United States tend to have higher rates of tuberculosis compared to states in the central part of the country, the region where our study was conducted [2,11]. Due to the conditions under which migration occurs, migrants are particularly susceptible to contracting communicable diseases and other factors that increase the risk of contracting tuberculosis, such as malnutrition [12,13,14]. Hence, HCWs in Mexican states bordering the United States have a higher risk of exposure to TB compared to those in central Mexico, as is the case with the participants in our study.

Our results suggest that men are more likely to be infected with TB. Although being male is a previously documented risk factor for TB infection [15], the mechanisms underlying this relationship remain unclear. An experimental study conducted in TB-resistant mice (strain C57BL/6) showed that the mortality rate and bacilli load during late disease were lower in females and neutered males. In addition, males had less lung inflammation than females and castrated males; hence, testosterone could play an immunosuppressive role in TB infection [16]. On the other hand, social roles and behaviors may be related to TB infection. Previous data show that in countries with a high burden of TB, smoking prevalence is higher among men [17], and several studies have hypothesized mechanisms linking smoking and alcohol consumption with tuberculosis risk [18]. Evidence has suggested that smoking may decrease the cytotoxic activity of natural killer cells, suppress T-cell function (both in the blood and lungs), and disrupt the mucociliary process that clears particles. Furthermore, cigarette smoke may promote the replication or persistence of phagocytosed mycobacteria by impairing the function of macrophages or dendritic cells [19,20,21].

On the other hand, excessive alcohol consumption impairs the immune function of the alveolar macrophage, the first line of defense against tuberculosis in the lower respiratory tract, and increases oxidative stress in the lungs, promoting the proliferation of tuberculosis bacteria [18,22]. Furthermore, epidemiological studies have suggested that chronic alcohol users are more likely to have recent TB transmission and to be part of a transmission cluster. While this increased transmission may be due to overcrowding in drinking establishments or to the fact that people who consume alcohol may be in close contact with others who are more likely to have TB, it could also be due to a higher mycobacterial burden in people with excessive alcohol consumption [22]. Another limitation was the lack of information regarding smoking and alcohol consumption habits. Rates of alcohol and tobacco consumption in the Mexican general population are higher among men [23,24]; therefore, the consumption of these substances could be different between women and men in our study population.

We found that direct contact with TB patients and more years of work experience increased the risk of TB infection; nevertheless, such findings were not statistically significant. Previous studies have shown similar but significant associations [10,25]. HCWs are regularly exposed to the environment of infected patients or have face-to-face contact with them; therefore, they are more likely to be infected with TB than the general population.

Our findings showed that physicians and stretcher-bearers were more likely to have LTBI than the nursing staff. Although these results are not statistically significant, they are consistent with those of a previous meta-analysis [25]. Nurses are at high risk of exposure to *MTB* because they have frequent contact with patients [26]. However, previous studies have shown that nurses have better knowledge of infection prevention guidelines, hand hygiene, and the correct use of personal protective equipment compared to other HWCs [27,28,29,30]. It has been documented that physicians have adequate knowledge of infection control, but their practices were often poor, especially regarding compliance with personal protective equipment [31,32,33,34]. Personal protective equipment is crucial to protecting healthcare workers from infectious diseases. Thus, the lack of adequate protection for physicians and frequent contact with TB patients may be a key factor determining TB infection among them.

### 4.1. Limitations

Our results have some limitations that need to be considered when interpreting them. This study was conducted at a single center; however, our findings may apply to other settings with similar TB rates. The cross-sectional design of this study did not allow for establishing the temporal sequence between the interest variables; thus, the estimated associations are not causal and should be interpreted cautiously.

While our logistic regression models were adjusted for potential confounders, it is essential to acknowledge that data on other relevant variables, such as alcohol and tobacco use, history of BCG vaccination, and comorbidities, were unavailable. Consequently, we cannot rule out the possibility of residual confounding affecting our findings. Both alcohol and tobacco use are known to impair the cellular immune response, mainly the function of macrophages and T lymphocytes, which are essential for containing *MTB*. This immune suppression facilitates the acquisition of *MTB* infection and its persistence in a latent state without clinical manifestations [35,36]. Furthermore, chronic diseases such as type 2 diabetes, present in approximately 18.3% of the Mexican adult population [37], may also compromise host defense mechanisms. Diabetes promotes a chronic inflammatory state and alters glucose metabolism in immune cells, which could impair their ability to respond effectively to TB. This dysfunction can also disrupt the production of key cytokines, such as interferon gamma (IFN-γ), crucial for controlling LTBI [38,39,40].

Although BCG vaccination history could act as a confounding factor when evaluating factors associated with LTBI, we believe this variable did not affect our results for two reasons. First, in the Mexican context, the BCG vaccine is routinely administered only to newborns, not adults. Second, the protective efficacy of BCG decreases over time; its immunological effect is estimated to last between 10 and 15 years. Therefore, childhood vaccination is unlikely to have interfered with interpreting the TST in the adult population evaluated in this study.

Finally, the sample size may have limited the statistical power of the study, which could partially explain the lack of statistically significant associations observed for several of the variables evaluated.

### 4.2. Implications for Practice

The findings of this study show that LTBI is a common problem among healthcare workers, even in settings considered to have a moderate incidence, such as Mexico. The detected prevalence of 16.7%, along with the higher risk observed in men and certain occupational groups such as physicians and orderlies, highlights the need to strengthen tuberculosis surveillance and prevention policies in hospital settings. These results suggest that occupational health strategies should prioritize the timely detection of LTBI through systematic screening protocols, especially among workers with greater exposure or with identified risk factors, such as male sex or direct contact with patients with active tuberculosis. Combining tuberculosis and immunological tests could increase diagnostic accuracy and facilitate informed clinical decision making.

Furthermore, these findings support the implementation of differentiated interventions tailored to professional roles, recognizing that not all healthcare workers are exposed to the same level of risk. Key strategies to reduce nosocomial transmission of *MTB* may include continuous infection prevention and control training, strengthening occupational epidemiological surveillance systems, and regular assessments of working conditions, particularly in high-risk areas such as inpatient care units. Finally, these findings may contribute to the development of public health policies in other healthcare settings with similar characteristics, aiming to protect both healthcare workers and patients through a preventive, equitable, and evidence-based approach.

## 5. Conclusions

Our findings indicate that approximately two in ten HCWs are affected by LTBI, with a higher likelihood observed among male workers. Preventing the progression to active TB and ensuring timely diagnosis of LTBI in HCWs are critical for protecting both individual health and broader public health systems. Despite growing recognition of TB as an occupational risk in healthcare settings, essential knowledge gaps persist, particularly regarding specific risk factors associated with LTBI among HCWs. Continued research is necessary to generate robust evidence to guide targeted prevention strategies and inform occupational health policies in similar contexts.

## Figures and Tables

**Table 1:** General characteristics of the study population.

Characteristics		Latent Tuberculosis		*p*-Value ^a^
	Total	No	Yes	
	n = 300 f (%)	n = 250 (83.3%) f (%)	n = 50 (16.7%) f (%)	
Sex				
Women	193 (64.33)	167 (86.5)	26 (13.5)	0.04
Men	107 (35.67)	83 (77.6)	24 (22.4)	
Age (years)				
<30	92 (30.67)	81 (88.0)	11 (12.0)	0.14
≥30	208 (69.33)	169 (81.3)	39 (17.7)	
Occupation				
Physician staffing	233 (77.67)	197 (84.5)	36 (15.5)	
Nursing staffing	46 (15.33)	37 (80.4)	9 (19.6)	0.43
Stretcher-bearer staffing	21 (7.00)	16 (76.2)	5 (23.8)	
Time of professional experience				
≤5 years	162 (54.00)	128 (85.2)	24 (14.8)	
>6 years	138 (46.00)	112 (81.2)	26 (18.8)	0.35
Contact with patients with tuberculosis at the hospital (within the last 5 years)				
No	95 (31.67)	82 (86.3)	13 (13.7)	
Yes	205 (68.33)	168 (81.9)	37 (18.1)	0.34
Contact with patients with tuberculosis outside the hospital (within the last 5 years)				
No	228 (31.67)	192 (84.2)	36 (15.8)	
Yes	72 (68.33)	58 (80.6)	14 (19.4)	0.46
Hospital Unit				
Outpatient care units	38	32 (84.2)	6 (15.8)	
Inpatient care units	262 (87.33)	218 (83.2)	44 (16.8)	0.87

^a^ Subjects were compared by latent tuberculosis status using Pearson’s chi-squared test or Fisher’s exact test for categorical variables.

## Data Availability

The data that support the findings of this study are openly available in Mendeley Data at http://doi.org/10.17632/fb2fcx9zgc.1.

## Supplementary Materials

The following supporting information can be downloaded at: https://www.mdpi.com/article/10.3390/diseases13060173/s1, S1: Crude logistic regression model for factors associated with LTBI.

## Abbreviations

The following abbreviations are used in this manuscript:

TB	Tuberculosis
LTBI	Latent tuberculosis infection
MTB	Mycobacterium tuberculosis
OR	Odds ratio
aOR	Adjusted odds ratio
CI	Confidence interval
