# Supplementary figures and images for: Prevalence and Associated Factors of Latent Tuberculosis Infection Among Healthcare Workers in a Mexican Tertiary Care Hospital
Source: Diseases. 2025 May 30;13(6):173. doi: 10.3390/diseases13060173 (PMC12191712; doi:10.3390/diseases13060173)

**Supplementary Material S1.** Crude logistic regression model for factors associated with LTBI.

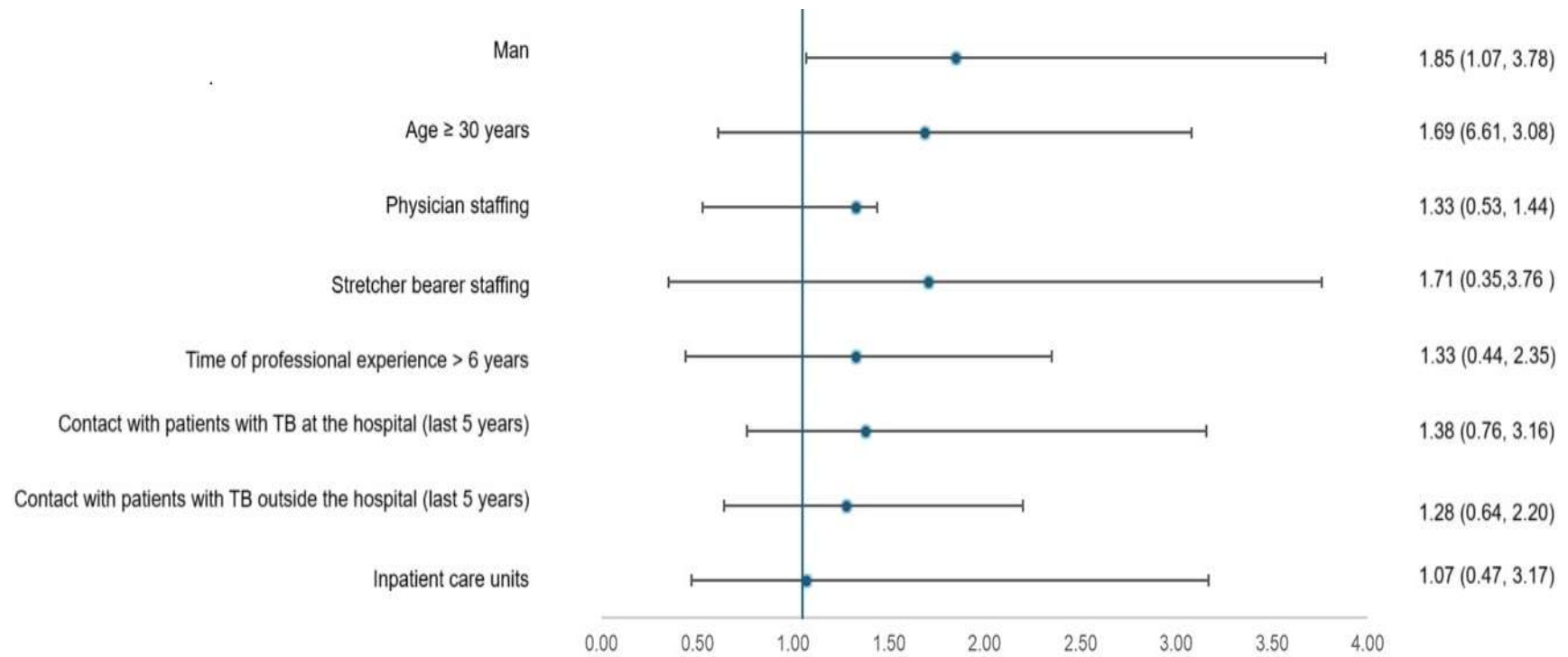

Supplement: Supplementary file 1 [file diseases-13-00173-s001.zip › diseases-3595515-supplementary.pdf]
